# Determinants of Maternal Health Care Utilization in Nigeria: a multilevel approach

**DOI:** 10.11694/pamj.supp.2014.17.1.3596

**Published:** 2014-01-18

**Authors:** Dorothy Ngozi Ononokpono, Clifford Obby Odimegwu

**Affiliations:** 1 Department of Sociology and Anthropology University of Uyo, Nigeria; 2 Demography and Population Studies Programme University of the Witwatersrand, Johannesburg, South Africa

**Keywords:** Delivery care, community, health facility delivery, maternal health care, maternal mortality, multilevel approach, primary sampling unit, utilization, Nigeria

## Abstract

**Introduction:**

Fourteen percent of maternal deaths globally occur in Nigeria. Low utilization of maternal health services for delivery may partially explain the high maternal mortality. The aim of this study was to examine the contribution of community factors in explaining variations in the use of health facilities for delivery in Nigeria.

**Methods:**

Our sample consisted of 17,542 women aged 15-49 years drawn from 2008 Nigeria Demographic and Health Survey, who had had their last birth in the five years before the survey. We employed multilevel analysis to identify community factors related to the use of delivery care.

**Results:**

In addition to several individual factors, region of residence was significantly associated with facility delivery. Women who lived in Northern Nigeria were less likely to deliver in a health facility than those who resided in the Southern part of the country. Residence in communities with a high proportion of women who had secondary and higher education significantly increased the odds of facility delivery whereas ethnic diversity was negatively associated with health facility delivery.

**Conclusion:**

Interventions aimed at promoting the use of health facility for childbirth should not only be implemented at the individual level but also tailored to the community level as interventions conceived without consideration for community context are likely to have limited impact. Increasing women's education in disadvantaged communities and region-specific interventions that increase access to health facilities are likely to have far-reaching impacts in reducing maternal mortality.

## Introduction

The increasing attention given to maternal health globally has concentrated on the reduction of maternal mortality. Although there has been a 47% decline in maternal deaths globally, the maternal mortality ratio is still unacceptably high. In high income countries, delivery is often a positive and fulfilling experience, but for many women in low-resource countries, delivery is associated with suffering, morbidity and in many cases maternal death [1]. With an estimated maternal mortality ratio of 500 per 100,000 live births, sub-Saharan Africa accounts for 56% of all maternal deaths globally [2]. Nigeria contributes 14% of global maternal deaths with a maternal mortality ratio of 630 per 100, 000 live births [3]. Although maternal mortality declined by 41% between 1990 and 2010 [2], Nigeria still ranks high in the list of countries with high maternal mortality rates. The high maternity rate has been attributed to inadequate use of maternal health care services [4].

Delivery in a health facility is associated with lower maternal and newborn mortality and morbidity rates compared with home delivery [5]. However, the 2008 Nigeria Demographic and Health Survey (NDHS) showed that only 38% of women delivered in a health facility [6]. Community perceptions of the quality of maternity health care, community beliefs about the importance of delivery in a health facility, knowledge of the benefits of having deliveries assisted by skilled health attendants, and place of last birth have been found to be associated with delivery in a health facility [7, 8]. Socioeconomic factors including husband's occupation, wealth status, and financial difficulty have been found to influence the utilization of maternity services both in Nigeria and elsewhere [9–12]. Adamu and Salihu also identified several socio-cultural factors, including illiteracy, husband's permission to use health services and purdah restrictions as barriers to women's use of hospital delivery in rural Kano, North west Nigeria [13]. Other studies in Nigeria found that region of residence, perceived quality of care, income, ethnicity and possession of health insurance were significantly associated with the use of delivery care service [14–17]. The decision to deliver in a particular health facility is also associated with proximity to the facility, cost and quality of care [18].

Several studies have identified individual and familial factors associated with the use of delivery care. However, most of these studies have shown an inconsistent pattern of association between the use of facility delivery and individual and household factors. For example, whereas Mpembeni and colleagues [19] found that delivery care is positively associated with maternal age, Aremu et al reported that maternal age was not significantly related to health facility delivery [17]. Further, although some studies have documented a positive effect of education on health facility delivery, some researchers have questioned the strong independent effects of education on the utilization of maternal health care services and have argued that factors such as place of residence and socioeconomic status interact to confound the strong effect of education on maternal health care behaviour [20].

As decisions to seek maternal health care are not solely dependent on individual characteristics, it is imperative to examine community conditions, such as social factors and the location of services [21], that can interact with individual preferences or choices to influence delivery care utilization. Researchers have argued that health promotion emphasizes the role of enabling environments and individual behaviour, therefore it is important to broaden the scope to other determinants of health, including community conditions [22]. The review of extant literature has shown that very few studies on maternal health care have examined factors at the community level. Most previous studies have rarely considered all the domains of the community. Examining community level factors related to place of delivery, for example ethnic diversity, may help in understanding aspects of communities that are important for policy manipulation. To address the identified research gaps, this study examined the relationship between community level factors and the use of health facility for childbirth in Nigeria.

## Methods

The data analyzed in this study were derived from the 2008 NDHS, a cross-sectional survey designed to provide information on population and health indicators at the national and state levels. The survey covered all 36 states and the Federal Capital Territory (Abuja). The sample frame for the survey was the list of Enumeration Areas (EAs) developed from the 2006 population census. The primary sampling units (PSU), which are referred to clusters, were selected from the lists of (EAs). The sample survey was selected using a stratified two-stage cluster design, made up of 888 (286 urban and 602 rural) clusters. Details about the sampling have been provided elsewhere [6]. In all, 33,385 women aged 15-49 years participated in the survey. The analysis reported in this study was limited to 17,542 women who had had their last delivery in the five years preceding the survey. Women who had more than one birth in the reference period were excluded from the analysis. Our choice of last birth is based on the assumption that information on maternal health care for the most recent birth is less subject to recall bias.

The dependent variable was place of delivery and was coded 1 if a woman delivered in a health facility and 0 if she delivered at home. We examined a number of individual/household variables including maternal age at last birth, education, religion, ethnic origin, occupation, women's autonomy, parity and socioeconomic status. Maternal age was calculated by subtracting the century month code (CMC) of the child's date of birth from the CMC of the date of birth reported by the respondent and categorized as: 15-24, 25-34 and 35-49. Education was categorized as: no education, primary, and secondary or higher. Religion was classified as Muslim, Christian and Traditional religion/others. Ethnic origin was categorized as Hausa (a merger of Hausa, Fulani and Kanuri based on geographical location and small number of Kanuri women in the sample), Igbo, Yoruba and Others (all the minority ethnic groups). Occupation was re-grouped into formal employment (professional/ technical/ managerial/ clerical/ sales/ services/ skilled manual workers), agricultural employment, unskilled manual workers and unemployed. Woman's autonomy was measured using a single variable assessing whether the respondent makes decisions on her own health care independently, jointly with her partner, or whether the partner or others make the decision. Parity was measured as the number of live births and categorized as 1-2, 3-4, 5 or more. Socioeconomic status was assessed using a household wealth index generated through Principal Component Analysis (from Factor Analysis) and based on household assets and amenities (e.g., type of flooring, water supply, electricity, radio, television, refrigerator and type of vehicle) [23].

We assessed four community level variables; place of residence (urban and rural), region of residence, community level education and ethnic diversity. Region of residence was categorized as: North Central, North East, North West, South East, South-South and South West. Community level education was measured as the proportion of women with secondary and higher education in the PSU. The measure was divided into three tertiles and categorized as low, medium and high. Ethnic diversity was defined as the proportion of women from different ethnic groups in the PSU [24]. The measure was divided into tertiles and categorized as low, medium and high. The community level variables were constructed by aggregating individual characteristics (educational attainment and ethnic origin) at the cluster level. The index woman was excluded while constructing the community level variables to reduce collinearity. A total of 886 clusters were represented in the study and each cluster or PSU was made up of a minimum of 80 households.

Bivariate tabulations were computed to identify the distributions of the outcome variables by selected background characteristics. The chi square test of association was used to test the statistical significance of these bivariate distributions. We estimated a multilevel model that assessed the relation of individual and community level factors (fixed effects) as well as community level random effects using Stata 11.1 software. Individual and household variables were considered as ‘individual level’ variables in the study. The average number of women in a household was 1.7; thus, the household was not large enough to be specified as a level of analysis.

Multilevel analysis was used to account for the hierarchical nature of the DHS data [25]. A two-level multilevel logistic regression model was estimated. The model consisted of two sub models at level 1 and level 2 (i.e., individuals (level 1) were nested within communities (level 2). A two-level multilevel model for a dichotomous outcome uses a binomial sampling and a logit link [26]. In level 1 model, the outcome variable Yij for individual i living in community j is written as follows:

1 $$\begin{array}{c} \text{Probability}\;(Y_{\text{ij}}=1|B)\;=\;\Phi_{\text{ij}}\\ \text{Level}\;1\;\text{variance}\;=\;\left[\Phi_{\text{ij}}\left(1-\Phi_{\text{ij}}\right)\right]\\ \text{Predicted log odds}\;\eta_{\text{ij}}=\;\text{log}\left[\Phi_{\text{ij}}/\left(1-\Phi_{\text{ij}}\right)\right]\\ \eta_{\text{ij}}=\;{ß}_{\text{oj}}+\Sigma {ß}_{\text{qj}}Xq_{\text{ij}} \end{array}$$

q = 1

Where: Φ_ij_ is the probability that the ith individual in the jth community take value “1” (“1” indicates that the event will occur); ß_0j_ is the level 1 intercept; ß_qj_ is level 1

coefficients. X_qij_ is level 1 predictor q for ith individual within jth community

The level 2 model, and can be expressed as follows:

2 $${ß}_{\text{qj}}\;=\;\gamma_{\text{q}0}+\;\gamma_{q1}W_{1j}+\;\gamma_{q2}W_{2j}+..+\gamma_{\text{qsq}}W_{\text{sqj}}+\;u_{\text{qj}}S_{q}=\;\gamma_{\text{q0}}+\;\sum \;\gamma_{\text{qs}}W_{\text{sj}}+\;u_{\text{qj}}$$

s = 1

Where

Γ_qs_ (q = 0,1,.....S_q_) are level 2 coefficients; Wsj are level 2 predictors and u_qj_ is level 2 random effects.

We estimated four models. The first model was an empty model containing no covariates, but decomposed the total variance into individual and community components. The second model included individual characteristics. The third model contained only the community characteristics and this allowed the assessment of the relation of the community variables to the outcome variable. The final model contained explanatory variables at both the individual and community levels and allowed the assessment of the net effect of community variables over and above the individual variables. The variables were retained in each of the models if the variance component was significant (p < 0.05) or if they were important demographic variables.

In all the estimated models, fixed effects were expressed as odds ratios (OR), while the random effects were expressed as variance partition coefficient (VPC) and proportional change in variance (PCV). The precision was measured by the standard error (SE). The VPC was calculated based on the linear threshold model method which converts the individual level variance from the probability scale to the logistic scale, on which the community level variance is expressed [27]. In this case, the individual level variance σ2e is equal to π2/3 (i.e., 3.29). The maximum likelihood was evaluated by integrating the random effects using the adaptive Gaussian quadrature (AGQ) [28] available in Stata; while the significance of the random effects were evaluated using the likelihood ratio (LR) statistics. AIC (Akaike information criterion) and the BIC (Bayesian information criterion) were used to test the goodness of fit of the models.

## Results

### Descriptive analyses

Forty-five percent of the sample was aged 25-34 years (Table 1).

**Table 1:** Percentage distribution of women by background characteristics

Characteristics	All women	
	%	n
**Maternal age at birth**		
15-24	36.9	6476
25-34	44.7	7847
35-49	18.4	3238
**Educational attainment**		
No education	45.4	7969
Primary	22.8	4004
Secondary	25.9	4542
Higher	5.9	1045
**Occupation**		
Unemployed	30.4	5312
Formal employment	41.4	7235
Agricultural employment	17.2	3005
Manual workers	10.9	1910
**Religion**		
Muslims	54.3	9482
Christians	44.0	7685
Traditional/Others	1.7	297
**Ethnic origin**		
Hausa/Fulani/Kanuri	39.6	6924
Igbo	11.6	2033
Yoruba	15.0	2627
Others	33.7	5887
**Women's autonomy (decisions over own health)**		
Wife alone	8.8	1450
Wife/husband	33.1	5477
Husband alone/Others	58.2	9634
**Parity**		
1-2	40.7	7144
3-4	32.7	5740
5 or more	26.6	4677
**Household wealth index**		
Poorest	23.1	4059
Poor	22.2	3898
Middle	19.0	3332
Rich	18.2	3187
Richest	17.6	3084
**Type of place of residence**		
Urban	30.2	5308
Rural	69.8	12253
**Region of residence**		
North Central	14.3	2516
North East	15.6	2745
North West	30.4	5337
South East	9.1	1599
South South	13.1	2303
South West	17.4	3061
**Community women's education**		
Low	42.6	7487
Medium	29.0	5097
High	28.3	4976
**Ethnic diversity**		
Low	41.1	7223
Medium	28.4	4984
High	30.5	5354

Forty-five percent of women had no education, while 31% had a secondary or higher level of education. Fifty-four percent of the women were Muslims and 44% were Christians. Almost a third (30%) of the women were unemployed. Forty percent of the women were Hausa/Fulani/Kanuri, 12% Igbo, and 15% Yoruba. More than half of the women (58%) reported that their husbands or other people have a final say over their own health. Fifty-nine percent of the women had given birth to more than two children. Seventy percent of the women lived in a rural area. About two in five (43%) women lived in communities with a low proportion of educated women and high ethnic diversity communities.

All explanatory variables were significantly associated with place of delivery at bivariate level. The bivariate results (Table 2) showed that a greater proportion of women aged 25-34 years (44%) delivered in a health facility than those aged 15-24 years (33%). While over 70% of women with secondary or higher education delivered in health facility only 11% of women with no education did so. A higher proportion of women in formal employment and Christian women delivered in a health facility compared with the unemployed and Muslims respectively. A lower proportion of women who had five or more live births and those who reported that their husbands or others make decisions on their own health care delivered in a health facility compared with women who had 1-2 live births and those who make decisions alone respectively. A key observation was the wide variation in health facility delivery observed among women from different ethnic groups. The proportion of women reporting that the most recent birth occurred in a health facility was higher for women of Igbo, Yoruba and minority ethnic origin compared with Hausa/Fulani/Kanuri women. Delivery in a health facility was higher for women who were in the richest wealth quintile. A lower proportion of rural than urban women delivered in a health facility. The use health facility for child delivery varied across regions, with a lower proportion of women from North West and North East delivering in a health facility. Women who lived in communities with a high proportion of educated women and a high proportion of women from different ethnic groups were more likely to deliver in a health facility compared with women who resided in disadvantaged communities.

**Table 2:** Percentage distribution of women by background characteristics, controlling for Place of delivery, Nigeria, 2008 DHS

Characteristics	Place of delivery	
	Health facility	P-value
***Maternal age at birth***		0.001
15-24	32.5	
25-34	44.4	
35-49	37.0	
***Educational attainment***		0.001
No education	10.9	
Primary	43.6	
Secondary	70.7	
Higher	91.4	
***Occupation***		0.001
Unemployed	28.1	
Formal employment	50.4	
Agricultural employment	31.9	
Manual workers	34.0	
***Religion***		0.001
Muslims	21.3	
Christians	60.9	
Traditional/Others	19.3	
***Ethnic origin***		0.001
Hausa/Fulani/Kanuri	9.3	
Igbo	79.7	
Yoruba	79.3	
Others	40.8	
***Women's autonomy (decisions over own health)***		0.001
Wife alone	59.3	
Wife/husband	53.6	
Husband alone/Others	26.3	
***Parity***		0.001
1-2	43.8	
3-4	39.3	
5 or more	30.1	
***Household wealth index***		0.001
Poorest	8.4	
Poor	17.5	
Middle	36.1	
Rich	61.9	
Richest	85.1	
***Type of place of residence***		0.001
Urban	65.3	
Rural	27.1	
***Region of residence***		0.001
North Central	43.2	
North East	14.0	
North West	9.3	
South East	77.8	
South South	51.8	
South West	77.7	
***Community women's education***		0.001
Low	9.3	
Medium	48.1	
High	73.2	
***Ethnic diversity***		0.001
Low	19.1	
Medium	59.6	
High	45.5	

### Multilevel analysis

The variation in health facility delivery across communities was significant (τ = 7.467, p = 0.001) (Table 3, model 1).

**Table 3:** Multilevel logistic regression odds ratio for the effect of individual and community factors on delivery care, Nigeria 2008 DHS

Characteristics	*Model 1 Empty model*	*Model 2 Individual variables*	*Model 3 Community variables*	*Model 4 Individual/Community variables*
		Odds Ratio	Odds Ratio	Odds Ratio
**Fixed effects *Individual characteristics Maternal age at last birth***				
15-24		1.000		1.000
25-34		1.142		1.093
35-49		1.394**	-	1.274*
***Educational attainment***				
No education		1.000		1.000
Primary		1.889***	-	1.518***
Secondary/Higher		4.133***		2.826***
***Religion***				
Muslims		1.000	-	1.000
Christians		1.243		0.955
Traditional/Others		0.445**		0.455***
***Ethnic Origin***				
Hausa/Fulani/Kanuri		1.000	-	1.000
Igbo		21.091***		4.699***
Yoruba		13.588***		2.920***
Others		3.871***		2.083***
***Occupation***				
Unemployed		1.000	-	1.000
Formal employment		1.220*		1.171*
Agric employment		1.034		1.044
Manual workers		1.021		1.058
***Women's autonomy (decisions over own health)***				
Wife alone		1.000		1.000
Wife/Husband		1.230		1.215*
Husband alone/Others		0.948		1.044
***Household wealth index***				
Poorest		1.000		1.000
Poorer		1.829***	-	1.478***
Middle		3.548***		2.121***
Richer		8.558***		3.599***
Richest		23.897***		7.222***
***Parity***				
1-2		1.000	-	1.000
3-4		0.683***		0.732***
5 or more		0.646***		0.707***
***Place of residence***				
Urban		-	1.000	1.000
Rural			0.385***	0.639***
***Region of residence***				
North Central		-	1.000	1.000
North East			0.178***	0.362***
North West			0.107***	0.215***
South East			2.200***	0.923
South South			0.611**	0.492***
South West			2.456***	1.451*
***Community women's education***				
Low			1.000	1.000
Medium			6.603***	2.710***
High			17.955***	4.012***
***Ethnic diversity***				
Low			1.000	1.000
Medium			0.727*	0.710*
High			0.544***	0.597**
***Random effects***	***Empty***	***Individual***	***Community***	***Individual/ Community***
***Variance* (SE)**	7.467*** (0.492)	1.933*** (0.774)	1.442*** (0.113)	1.118 ***(0. 097)
(VPC) =ICC (%)	69.4	37	31	25.4
(PCV) (%)	Reference	74.1	80.7	85
***Log-likelihood***	-7376.9092	-5957.9257	-6844.3507	-5830.1125
***Model fit statistics***				
***AIC***	14759.8	11961.9	13714.7	11726.2
***BIC***	14783.2	12139.4	13816	11980.9

Note: Only variables with a significant (p < 0.5) variance component were included in the models. The empty model contains no variables but partitions the variance into two component parts. SE = Standard error, VPC= Variance Partition Coefficient, PCV = Proportional Change in Variance. AIC = Akaike Information Criterion, BIC = Bayesian Information Criterion.

*** Significance level p <0.001

** Significance level p < 0.01

* Significance level p < 0.5

The variance partition coefficient as shown by the estimated intercept component variance was 69.4%. This is the variability in the outcome variable attributed to the community level.

Model 2 contained only the individual level variables. Results showed that maternal age was significantly associated with health facility delivery, with older women (35-49 years) having higher odds of delivering in a health facility compared with younger women aged 15-24 years. There was no statistical significant association between a woman's autonomy and delivery in a health facility. Women with secondary/higher education had 4 times greater odds of delivery in a health facility than women with no education. There was no significant difference between Muslims and Christians in place of delivery. Women from Igbo, Yoruba and others (minority ethnic groups) were more likely to deliver in a health facility compared with Hausa/Fulani/Kanuri women. Similarly, compared with the unemployed, women in formal employment had higher odds of delivering in a health facility. Increasing socioeconomic status was associated with greater odds of having a delivery in a health facility. Parity was negatively associated with delivery care. As parity level increased, women were less likely to deliver in a health facility compared with low parity women (women with 1-2 live births). Compared to the empty model, the variation in health facility delivery was significant across communities (τ = 1.933, p < 0.001). The intra-community correlation was 37% indicating the variability in the outcome variable.

In model 3, all the community variables were significantly associated with delivery care. Rural women had 61% lower odds of delivering their baby in a health facility compared with urban women. Interestingly, the odds of delivering a child in a health facility were 2.5 times and 2.2 times higher for women from South West and South East respectively, compared with those from North Central. However, women from North East and North West had lower odds of delivery in a health facility than those from the North Central region. Women who lived in communities with a high proportion of educated women were more likely to use delivery care compared with women who resided in disadvantaged communities. However, living in communities with a high proportion of women from different ethnic groups was associated with lower odds of delivery in a health facility. In comparison to model 2, the variance in health facility delivery across communities was significant (τ = 1.442, p < 0.001). The intra-community correlation estimate reduced to 31%. The inclusion of the community level factors reduced the community level variance from 1.933 to 1.442 (p < 0.001).

Controlling for individual/household and community variables in the final model (model 4), maternal age at last birth, education, religion, occupation, ethnic origin, woman's autonomy, parity and household wealth index were found to be significantly associated with health facility delivery. The pattern of association remained the same as in model 2. However, the inclusion of the community variables attenuated the odds for educational attainment, ethnic origin and household wealth index. Interestingly, women's autonomy was significantly associated with health facility delivery unlike in the model without the community level variables.

The association between delivery care and community variables yielded interesting results. The odds of having a health facility delivery were lower for women in South west and South east compared with women in North Central, although the association was no longer statistically significant for South east. Further, inclusion of individual factors attenuated the odds for community women's education compared with the model that included only community level variables. The community level variance was significant (τ =1.118, p < 0.001). The intra-community correlation decreased to 25.4% suggesting that the inclusion of the community variables improved the overall explained variance in the use of delivery care compared with model 3. Moreover, the smaller values of AIC and BIC indicated that model 4 was a better explanatory model.

## Discussion

Study findings demonstrated the significant association between community level factors and delivery in a health facility. Importantly, we found a strong association between community level education and delivery in a health facility. The finding that the likelihood of delivering a baby in a health facility was higher for women from communities with a high proportion of women with secondary/higher education compares well with findings elsewhere [5, 29]. These results suggest that in communities where educational attainment is high, women may have more material resources and autonomy to access health care services. The strong association of community women's education points to the need to increase women's education particularly in disadvantaged communities.

Surprisingly, ethnic diversity showed a negative significant association with delivery care. Women in communities with a high proportion of women from different ethnic groups had a lower likelihood of delivering their baby in a health facility. The nature of this relationship is unclear, but could reflect “the heterogeneity and social and ecological settings in Africa” [30], which are barriers to utilization of maternal health care services. Furthermore, the negative association between ethnic diversity and poor health outcomes could be explained in relation to the problems of collective action faced by many heterogeneous communities in Africa [31].

Region of residence was significantly associated with facility delivery. Women from the North East and North West were less likely to deliver in a health facility. Regional differences in the use of maternal health care could reflect disparities in socio-economic development, as more health care services are located in the Southern regions of Nigeria than in the Northern regions [6]. The finding that women from Igbo, Yoruba, and Northern/Southern minority ethnic groups had a higher likelihood of using health facility for childbirth compared with Hausa/Fulani/Kanuri women, underscores the complexity of forces in operation among the different ethnic groups in a culturally-diverse society like Nigeria [25].

With respect to individual level variables, educational attainment, occupation, ethnic origin, a woman's autonomy, household wealth index, parity and religion were significantly associated with delivery care. These results are consistent with studies other studies in developing countries [2, 32, 33]. Educational attainment, occupation and household wealth are markers of economic resources which empower women to take control of their own health and facilitate easy access to quality maternal health care [25] Results indicate that women who reported making joint decisions with their partners were more likely to deliver in a health facility. This finding suggests the need for programmes to improve women's status and autonomy, and also involve women's partners in maternal health programmes so as to educate them on issues regarding appropriate health care seeking during delivery [29].

The likelihood of having a delivery in a health facility decreased consistently as number of children ever born increased. Presumably, this may reflect the assumption that women of higher parity are less likely to deliver in a health facility either because of their maternity experiences or because having a large family size means having fewer resources (both time and money) available to seek maternal health care [2, 34]. Therefore there is need for policy that encourages small family sizes or women to access delivery care services for every pregnancy.

Study findings should be interpreted in light of some limitations. First, the study used primary sampling units as a proxy for the community. As observed, using the DHS PSU as the community may bias results because of selection effects [35]. Second, some important factors known to influence delivery care service utilization (e.g., distance to a health facility) were not included in our analyses due to data limitation. The omission of these important confounders may have biased the estimates of the measured variables in this study. Third, the community variables were constructed by aggregating the individual level characteristics at the community level and this may be associated with problem of making inferences at a higher level based on information from data collected at the individual level [35]. Fourth, the 2008 NDHS data were collected retrospectively and may be associated with recall bias. Finally, the cross-sectional nature of the survey does not allow for cause-effect inferences. However, the study remains significant. The study is based on a large nationally-representative population-based survey whose findings are relevant for comprehensive national policy initiatives. The study is also relevant in identifying aspects of the community that are important for interventions. However, qualitative research is needed to adequately understand the association between the cultural identity factors (ethnic origin and region of residence) and place of delivery.

## Conclusion

Community factors were significantly associated with the use of maternal health care. Interventions aimed at promoting the use of health facilities for childbirth should not only be implemented at the individual level but tailored to the community level as interventions conceived without consideration for community context are likely to have limited impact. To close the gap in facility delivery, community and regional specific interventions that allow equitable distribution of maternal health care services should be implemented. Importantly, there is need for interventions that explore the most effective ways to raise women's status in terms of education and socio-economic status in disadvantaged communities.
